# Insights on the User Experience and Feasibility of an Electromyography-Driven Exergame Combined With Blood Flow Restriction for Strength Training in Hospitalized Older Adults: Mixed Methods Randomized Controlled Feasibility Study

**DOI:** 10.2196/69400

**Published:** 2025-06-10

**Authors:** Ruben Debeuf, Reinhard Claeys, Margo Berlanger, Myrthe Bunt, Aziz Debain, Daan De Vlieger, Matthias Eggermont, Mahyar Firouzi, Stefania Guida, Katarína Kostková, Siddhartha Lieten, Lubos Omelina, Silvia Zaccardi, Bart Jansen, Eva Swinnen, David Beckwée

**Affiliations:** 1 Rehabilitation Research Group, Department of Physiotherapy, Human Physiology and Anatomy, Faculty of Physical Education and Physiotherapy, Vrije Universiteit Brussel, Laarbeeklaan 121, Brussels, 1090, Belgium, 32 24774326; 2 Center for Neurosciences (C4N), Vrije Universiteit Brussel, Brussels, Belgium; 3 Brussels Human Robotic Research Center (BruBotics), Vrije Universiteit Brussel, Brussels, Belgium; 4 Geriatrics Department, Universitair Ziekenhuis Brussel, Brussels, Belgium; 5 Department of Rehabilitation Sciences, Ghent University, Ghent, Belgium; 6 Brain, Body and Cognition, Department of Psychology and Educational Sciences, Vrije Universiteit Brussel, Brussels, Belgium; 7 Unit of Clinical Epidemiology, Istituto di Ricovero e Cura a Carattere Scientifico Ortopedico Galeazzi, Milan, Italy; 8 Department of Electronics and Informatics, Vrije Universiteit Brussel, Brussels, Belgium; 9 Research Group Movement Antwerp (MOVANT), Department of Rehabilitation Sciences and Physiotherapy, University of Antwerp, Antwerp, Belgium

**Keywords:** exergames, older adults, blood flow restriction, feasibility, user experience

## Abstract

**Background:**

Hospitalized older adults often spend prolonged periods of time bedridden, leading to decreased muscle strength and function. To tackle this, rehabilitation aims to keep patients active and train affected muscles. Exergames have proven to be effective in the rehabilitation of different patient populations and offer a motivating solution to combat inactivity associated with hospitalization. Furthermore, blood flow restriction (BFR) is effective in therapy for weakened patients, so combining BFR and exergames might be promising.

**Objective:**

As part of an iterative process of user-centered development, this mixed method study investigates the acceptability and feasibility of the Ghostly game as a stand-alone added therapy or combined with BFR in strength training of hospitalized older adults.

**Methods:**

A mixed methods study was conducted on 15 hospitalized older adults. Participants were randomized into 3 groups and received daily interventions from the moment they were included in the geriatric ward, until discharge from the hospital. The Ghostly group received daily conventional therapy with the Ghostly game as added therapy, the Ghostly + BFR group received daily conventional therapy with Ghostly in combination with BFR as added therapy and last, the control group received daily conventional therapy with dose-matched isometric exercises as added therapy. The primary outcome, user experience, was assessed before discharge from the hospital using the Usefulness, Satisfaction, and Ease of Use questionnaire and through expert observations. Clinical outcomes such as muscle strength, muscle architecture, and segmental body composition were assessed at baseline and before discharge from the hospital to test the feasibility of the research protocol in preparation for future randomized controlled trials.

**Results:**

A total of 15 hospitalized older adults (11 female participants, 73.33%) were included in this study with an average age of 84.53 (range: 78‐94) years. Participants received an average of 3.47 (range: 3‐5) intervention sessions after transferring to the geriatric ward of the hospital. Results on user experience revealed high scores on all subcategories of the Usefulness, Satisfaction, and Ease of Use questionnaire (usefulness: 78.93%, ease of use: 82.99%, ease of learning: 85.36%, and satisfaction: 87.55%). Furthermore, expert observations identified issues with color contrast, reaction time speed, and the need to tailor the game to accommodate the diverse requirements of different patient populations. All outcomes and procedures were found feasible for a future randomized controlled trial.

**Conclusions:**

This mixed methods study combines the innovative aspects of an electromyography-driven exergame with strength training principles of BFR and reveals the acceptability and feasibility of the Ghostly game as a stand-alone added therapy modality for strength training in hospitalized older adults and in combination with BFR. Future improvements of the exergame could focus on addressing expert-identified issues, including optimizing color contrast, adjusting reaction time speeds, and tailoring the game to meet the needs of different patient populations.

## Introduction

Hospitalization in older adults poses a high risk for functional decline due to acute inflammatory conditions, prolonged periods of bed rest, and reduced activity [1,2]. They are especially prone to inactivity and sedentary behavior during nontherapy time, as time spent in scheduled rehabilitation is often limited [3,4]. To engage older adults in rehabilitation, especially during nontherapy time, it is important to increase patient motivation to train. Evidence already showed that even short periods of disuse (ie, 10 d) induce a decline in rates of muscle protein synthesis and a rise in muscle protein breakdown, which, in turn, results in a rapid rate of muscle atrophy over the first few days of disuse [5]. Furthermore, in an older population, we see a reduced capacity to regain lost muscle tissue during recovery from disuse [5]. Moreover, this loss of functional independence, often attributed to muscle mass loss, is known as hospital-associated disability and affects 30% of hospitalized adults aged 65 years and older [6,7]. Recognizing this reduced functional capacity is detrimental to implementing evidence-based interventions to preserve and potentially improve outcomes in hospitalized older adults. Strategies to mitigate these effects of disuse include targeted physical activity [8]. However, older adults, especially those who are bedridden, are often unable to perform high-load, dynamic functional exercises, further limiting recovery potential. Nonetheless, low-load interventions, such as isometric strength exercises in combination with blood flow restriction (BFR), might provide a feasible solution for bedridden patients to commence rehabilitation in an early stage of hospitalization [9-11]. BFR entails the partial occlusion of blood flow towards the exercising limb, which results in a partial inflow of arterial blood and complete restriction of venous outflow [12,13]. This novel strength training modality has proven to have beneficial effects on muscle strength compared with conventional low-load strength training in older adults [14]. Evidence on the safety of BFR in adults aged 65 years and older shows that low-load BFR training increases heart rate and blood pressure (systolic and diastolic) to levels similar to those observed during conventional high-load training, indicating no greater risk [15]. However, to minimize the risk of adverse events and ensure safe training, contraindications such as poor circulatory health, open fractures, and infections should be carefully considered [12]. Despite the evidence on the safety and effectiveness of BFR, motivation to exercise remains an important limiting factor.

A novel therapy modality that has proven effects on motivation is exergames for rehabilitation. Exergames combine exercise with the motivational aspect of interactive video games [16]. In older adults, it was found that exergames have a positive effect on physical function [17]. Additionally, Rytterström et al [18] reviewed the feasibility and practical implications of exergames in this population and reported that older adults found exergaming physically and cognitively demanding, yet enjoyable. Focusing on physical function in older adults, the systematic review and meta-analysis of Pacheco et al [19] showed evidence that exergames can improve balance and mobility and motivate patients to keep performing the exercise. However, the combination of BFR and rehabilitation technology, such as exergames, is not addressed in the existing literature.

To provide a solution to the earlier-mentioned issues of inactivity and low motivation to train in nontherapy time and the need for low-load strength training in bedridden patients, we developed a prototype version of an electromyography (EMG)-driven exergame, the Ghostly game. The Ghostly game is a novel isometric strength training tool for rehabilitation, cocreated based on the principles of user-centered design. The goal of user-centered design is to incorporate the knowledge from different experts in the field, as well as insights from the end users to maximize adoption, user experience, and clinical relevance [20]. The development process of the Ghostly game commenced in 2016 and is marked by progressive iterations and advancements from its inception through various stages of development, testing, and refinement. The main goal for the Ghostly game throughout this entire process remains to incorporate evidence-based guidelines on strength training and rehabilitation in the game. As mentioned earlier, isometric strength training can prove relevant in the early stages of hospitalization [10,11], so guidelines on isometric strength training were incorporated in the level design to ensure effective state-of-the-art strength training. To further increase the potential effectiveness, it was decided to combine the Ghostly game with the evidence-based principles of BFR training to ensure positive effects during low-load isometric strength training.

Various game-based EMG biofeedback systems for rehabilitation have already been investigated, but evidence on clinical effectiveness remains limited [21,22]. A study by Garcia-Hernandez et al [23] describes an EMG-controlled exergame to enhance grip strength. The exergame used in this study requires users to contract their muscles at 100% of their maximal voluntary contraction (MVC) to complete the different goals set in the game. A total of 16 healthy participants completed 2 weeks of strength training using the exergame, which resulted in a significant increase in grip strength. Additionally, Garcia-Hernandez et al [23] reported significantly higher intrinsic motivation in participants who trained with the exergame compared with participants performing conventional therapy. Most importantly, the results demonstrated that the exergames group adhered significantly better to the work-rest time proposed in the study, and the MVC was significantly higher in the exergames group compared with the conventional therapy group [23]. Enciso et al [24] focused on exergaming for people with lower limb impairments who use a wheelchair. The exergame “Workout on Wheels” is aimed at encouraging and facilitating exercise at home. Three games were incorporated within the app to strengthen the muscles of the upper limb using surface EMG sensors to register muscle contractions. Enciso et al [24] tested the feasibility of this exergame in 4 participants with varying levels of spinal cord injury and found that the participants perceived the game as highly useful and easy to use. Additionally, the results from the open-ended questionnaire revealed that the exergames helped participants exercise animatedly and engagingly and motivated them to work out. No clinical outcomes were detected for this study [24]. Both of these studies highlight the potential of an EMG-driven exergame but also indicate the need for more research into its effectiveness in rehabilitation.

This study investigates an EMG-driven exergame combined with evidence-based principles of BFR training for strength training in a hospital environment. As an early part of a larger iterative process of user-centered development of rehabilitation technology [20], the goal was to gather relevant insights on the needs and barriers of end users to maximize user experience and effective adoption in rehabilitation. The study focused on the development of the Ghostly game, as well as the innovative aspects of Ghostly combined with BFR. Additionally, the feasibility of the research protocol in preparation for a larger randomized controlled trial (RCT) was assessed. The final aim of this study is to evaluate the data gathered to determine whether an updated version of the Ghostly game is needed.

## Methods

### Study Design

This mixed method study used an RCT design with 3 study arms and focused on gaining insights into the acceptability and feasibility of the Ghostly game as an added therapy modality on top of the conventional therapy in strength training of hospitalized older adults. Qualitative and quantitative data were collected during and after the intervention period in which participants received their daily conventional therapy, with an added therapy modality based on group allocation. Patients were randomized into 3 groups using sealed envelopes. The first experimental group received the conventional therapy supervised by the physiotherapist of the geriatric ward, and the Ghostly game as added therapy supervised by the researchers. Similarly, the second experimental group received conventional therapy and the Ghostly game combined with BFR as an added therapy. Last, the control group received their conventional therapy and the added therapy of isometric exercises dose-matched to the Ghostly game in terms of repetitions, sets, and interset rest. All exercises were guided by the researchers and solely focused on both quadriceps muscles. The researchers instructed the participant to keep the lower limb fixed on the bed throughout the entire exercise session to ensure activation of the quadriceps muscle without external fixation of the lower limb. In our approach, we prioritized eliciting strong and consistent contractions of the quadriceps femoris. However, we did not implement any specific containment or isolation procedures to prevent the cocontraction of other lower limb muscles. This decision reflects our intent to maintain a more functionally relevant setup, as such cocontractions naturally occur during daily activities. The quadriceps were selected as the primary focus due to their critical role in functional movements such as standing, walking, and stair climbing, as well as their relatively large muscle mass compared with other lower limb muscles. Participants received daily interventions from the moment they entered the geriatric ward and were deemed fit for training with the Ghostly game by the health care professionals of the ward until discharge from the hospital. Data were collected at baseline and before discharge from the hospital.

### Ethical Considerations

The study was conducted according to the guidelines of the Declaration of Helsinki [25], approved by the ethics committee of the University Hospital of Brussels (Eudamed: CIV-22-02-038967), and the details of the protocol were registered a priori (registration number: NCT05258500). All participants read and signed the informed consent form before inclusion in the study. All study data were anonymized to remove identifiable information of the participants and ensure privacy and confidentiality.

### Participants

Patients were assessed for study eligibility by the physiotherapist of the geriatric ward on the day they were transferred to the geriatric ward and were included in this study if they met all of the following criteria: hospitalized, 65 years or older, and unable to perform 14 repetitions on the 30 seconds sit to stand test [26]. Patients were excluded from this study if they were unable to understand the basic instructions of the Ghostly game or had other severe conditions that prohibited them from performing lower limb strength training in combination with BFR. Relevant demographic information was collected from the medical files of the participants before discharge from the hospital.

Since the study was a feasibility study, no a priori sample size calculations were performed, and a sample of 15 participants was chosen based on practical considerations and the number of participants needed to reasonably evaluate feasibility goals and gather meaningful data on user experience [27].

### Intervention

#### Ghostly

**Figure 1.**
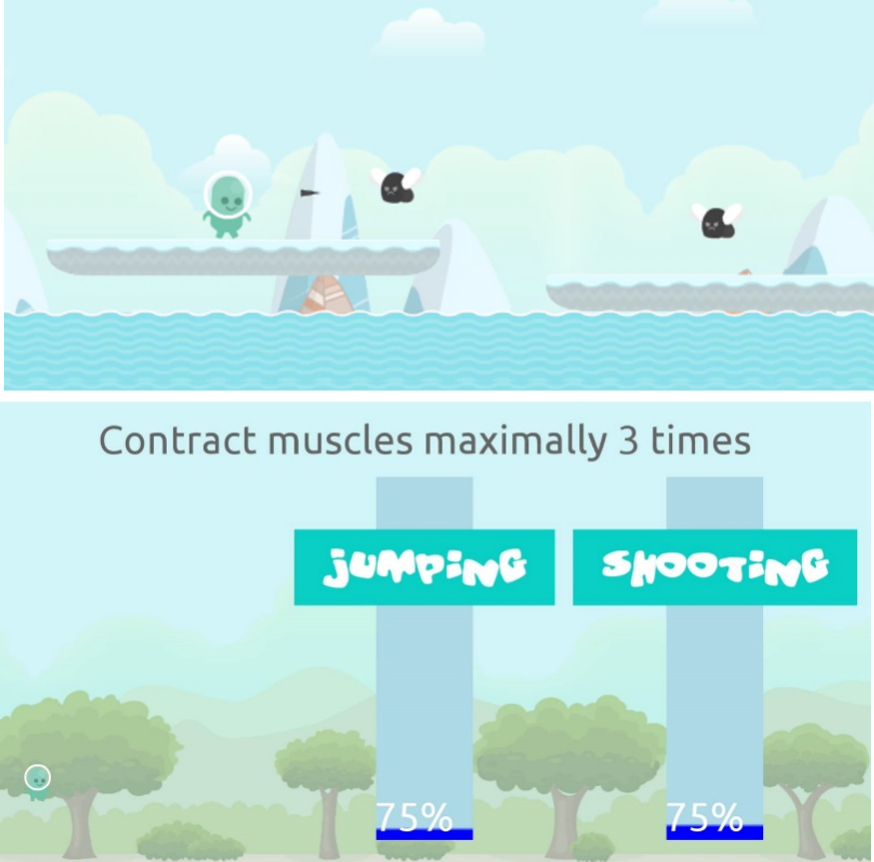
The Ghostly game and the assessment level (showing the intensity sliders) to set the training intensity of the game based on maximal voluntary contraction.

**Figure 2.**
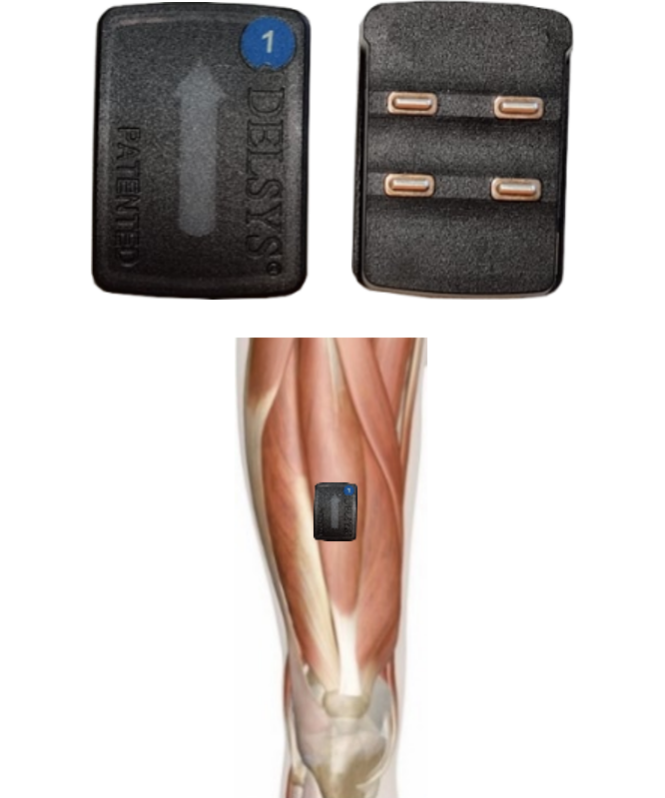
The surface electromyography sensors used to control the main character, and the placement of the surface electromyography sensors over the belly of the quadriceps muscle.

The Ghostly game (Figure 1) is an EMG-driven exergame in which the main character is controlled through muscle contractions measured by 2 surface EMG sensors (Figure 2) placed on the skin over the targeted muscles using double-sided adhesive tape. The Ghostly game was developed as an individually tailored strength training modality for bedridden patients that adheres to evidence-based guidelines on strength training. To complete levels in the Ghostly game, the participant needs to lead the main character to the end of the level by shooting enemies (ie, contraction of the muscle of one limb) and jumping over obstacles (ie, contraction of the muscle of the other limb). The duration of the contraction is based on the evidence-based guidelines that have been implemented in the in-game animations, which last on average one second. All levels were designed to require at least 12 contractions with each limb to reach the end. This way, as participants were asked to complete 3 levels, they performed 3 sets of 12 repetitions [28].

Since training at one’s personal maximal capacity is a priority, the Ghostly game incorporates a baseline assessment level (Figure 1) in which the MVC of the user is measured while the participant remains in a supine position in bed with the legs extended. Two surface EMG sensors were placed over the belly of the target muscles of the participant by the researcher. First, the assessment level, consisting of 3 maximal contractions, with each contraction lasting 3 seconds and with 90 seconds of rest between each contraction, needs to be completed [29]. After the first maximal contraction, an MVC value is set to match the intensity of this first contraction. If subsequent contractions exceed the MVC value, the value is raised accordingly. Based on this MVC value, the intensity at which the user must contract the muscles to control the main character of the game is set to match an intensity of 75% of MVC [30,31] as this has been proven to increase muscle hypertrophy [30] and muscle strength [31] in isometric strength training. After completion of the assessment level, a training level was selected, and the participants started playing the game.

As the target population for this feasibility study consists of bedridden patients who benefit from lower limb isometric strength training in preparation for more functional gait training, the sensors were placed over the belly of the quadriceps muscles of both legs (Figure 2) following the SENIAM guidelines [32].

#### Blood Flow Restriction Therapy

The second therapy arm of this study involves the addition of BFR therapy to the Ghostly game. Cuffs (width: 10 cm, length: 58 cm) were placed over the proximal part of both lower limbs to partially occlude the blood flow towards the quadriceps muscles. To achieve the desired level of occlusion to the target muscle, a percentage of the arterial occlusion pressure (AOP) was used, measured by the SmartCuffs pro system (Smart Tools). AOP is defined as the amount of pressure required to completely cease arterial blood flow toward the limb [33]. To determine AOP, the cuffs are filled with air until a pressure is reached at which no arterial blood pressure is registered by the SmartCuffs pro system. This pressure is then set as the AOP, and all air within the cuffs is automatically released. Afterward, the cuffs are reinflated until a certain percentage of the personalized AOP is reached. As pressures ranging from 40% to 80% of AOP have been proven to be effective in strength training [33,34], and with the goal of minimizing discomfort for the participants, an occlusion pressure equal to 50% of AOP was used in this feasibility study. A comprehensive overview of the complete intervention is given in Multimedia Appendix 1.

### Outcome Measures

#### Primary Outcome: User Experience (USE)

The primary outcome of this study was the user experience of hospitalized older adults after playing the Ghostly game, which was assessed before discharge from the hospital using the Dutch and French versions of Usefulness, Satisfaction, and Ease of Use (USE) questionnaire [35]. This 30-item questionnaire is separated into 4 subcategories: usefulness, ease of use, ease of learning, and satisfaction. Each item is scored on a 7-point Likert scale, with a score of 1 indicating “strongly disagree” and a score of 7 indicating “strongly agree.” A high score on the USE questionnaire indicates good usability of the device [36]. Results are reported as a mean percentage of the total score of the USE questionnaire, as well as a mean percentage of the scores of each subcategory. The USE questionnaire shows high reliability and validity, in line with other usability questionnaires [36]. Both the Dutch and French versions of this questionnaire have been used in research previously [37-39]. On top of this, open questions were added to the questionnaire that focused on relevant changes that should be made to the Ghostly game.

#### Secondary Outcome: End User Feedback and Expert Observations

To ensure all data on user experience and important barriers were gathered, a structure similar to think-aloud protocols was used in which expert observations and patient feedback were documented through field notes [40,41]. A think-aloud protocol aims to gather information on how the participants interact with the intervention by asking them to think aloud as a means of capturing the problem-solving process and refining the intervention to be more effective [41]. In this study, the researchers and experts observed and noted the thoughts and experiences of the participants while they interacted with the Ghostly game.

Patient feedback and real-time feedback while engaging with the Ghostly game, either as a stand-alone added therapy or combined with BFR, were collected throughout all intervention sessions. Comments and recommendations were documented using field notes and subsequently compared across participants.

Expert observations by different health care professionals (ie, physiotherapists and physicians), engineers, and researchers were conducted at various time points throughout the study to identify key barriers and elements within the game that require a change to maximize user experience. The experts followed multiple participants across different sessions to gather data on both difficulties during the initial learning phase, as well as difficulties in later sessions when participants had become familiar with the game. Experts focused on game design, difficulties while playing, relevance for rehabilitation, etc. Furthermore, the experts prioritized gathering input on the feasibility of applying and explaining the Ghostly game and BFR therapy in relation to future unsupervised training with the game.

#### Feasibility of the Intervention and Clinical Outcomes

The feasibility of the clinical study protocol was assessed as a preliminary step in preparation for a fully powered RCT. For this, the following clinical outcomes were measured: muscle strength of the quadriceps muscle, muscle architecture of the rectus femoris muscle, and body composition of both lower limbs at baseline (T0) and before discharge from the hospital (T1). Muscle strength was measured using the MicroFET 2 handheld dynamometer (Hoggan Health) and the 30-second sit-to-stand test [26]. Muscle architecture (cross-sectional area [CSA], muscle thickness, and subcutaneous fat) was measured using the Viamo sv7 ultrasound device (Canon Medical Systems, Japan). And last, body composition was measured using the Quadscan 4000 bio-impedance analysis device (Bodystat, Douglas). Because of the aim of the Ghostly and BFR interventions (targeting muscle mass loss), we were specifically interested in appendicular lean mass (ALM). To calculate ALM, the following formula was used:


$$ALM=0.827+\left(0.19*(\frac{length^{2}}{Impedance50Hz})\right)+\left(2.101*sex\right)+\left(0.079*weight\right)$$

where sex equals 0 for female participants and 1 for male participants [42].

The feasibility criteria for the success of the study protocol were for all participants to be able to complete the intervention. Full adherence to the study protocol is reached when participants complete daily interventions from baseline until discharge from the hospital. Furthermore, 80% or more of participants should complete all primary and secondary measurements. Last, if adverse events occurred, they were noted. The premises of the threshold were set to ensure sufficient therapy adherence and compliance in preparation for the future larger RCT.

### Statistical Analyses

The primary outcome measures were analyzed using Microsoft Excel (Microsoft Office 2024). Mean scores, SDs, and medians were calculated for each subcategory of the questionnaire. All clinical outcomes were statistically analyzed using SPSS statistics software (version 28; IBM Corp). Descriptive statistics were used to determine the baseline values of the participants. Mean difference and 95% CIs were calculated. Last, box plots of the most relevant clinical outcomes were created to visualize the distribution of the data.

## Results

### Participant Characteristics

**Figure 3.**
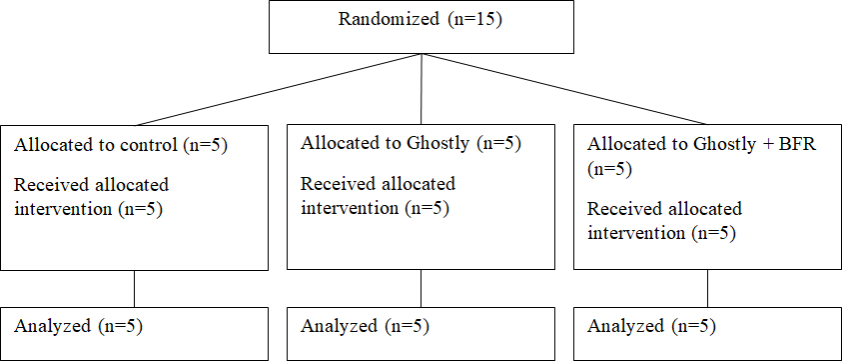
CONSORT diagram for the 3 intervention groups. BFR: blood flow restriction.

In total, 15 participants (11 female participants, 73.33%), 5 in each study arm, were included in this feasibility study with an average age of 84.53 (range: 78‐94) years (Figure 3). Participants received an average of 3.47 (range: 3‐5) intervention sessions after transferring to the geriatric ward of the hospital. Table 1 gives an overview of the baseline characteristics of the participants.

**Table 1.** Baseline characteristics of all participants.

Baseline characteristics of all participants (n=15)	Values
Age (years), mean (SD)	84.53 (4.53)
Sex, n (%)	
Female	11 (73.33)
Male	4 (26.67)
Number of intervention sessions, mean (SD)	3.47 (0.72)
30-second sit-to-stand (repetitions), mean (SD)	3.33 (2.55)
Quadriceps muscle strength (Newton), mean (SD)	75.75 (20.92)
Rectus femoris muscle thickness (cm), mean (SD)	0.87 (0.27)
Rectus femoris muscle cross-sectional area (cm²), mean (SD)	2.89 (1.26)
Lower limb subcutaneous fat (cm), mean (SD)	0.85 (0.52)
Lower limb lean mass (kg), mean (SD)	24.10 (5.20)

### Primary Outcome: User Experience

**Figure 4.**
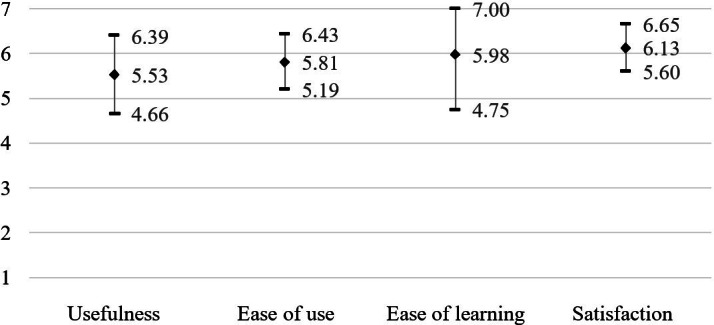
Mean scores for each subcategory of the Usefulness, Satisfaction, and Ease of Use questionnaire. Total mean scores for each subcategory were transformed to fit a 7-point Likert scale. The dot indicates the mean score for each subcategory, and the whiskers represent the mean score plus and minus 1 SD. A score of 7 indicates participants highly agree with the statement, and a score of 1 indicates that participants highly disagree with the statement.

After completion of the intervention, all 10 participants who trained with the Ghostly game (study arms 1 and 2) completed the USE questionnaire. The results (Table 2 and Figure 4) show an overall high mean score on the USE questionnaire (83.29%). Dividing the results into the subcategories shows a mean score of 78.93% for usefulness, 82.99% for ease of use, 85.36% for ease of learning, and 87.55% for satisfaction, indicating a high mean score across all subcategories. When separating the results of the Ghostly group and the Ghostly + BFR group (Multimedia Appendix 2), an overall high mean score on the USE questionnaire can be found for both groups (Ghostly: 86.38%; Ghostly + BFR: 80.19%), implying no influence of BFR on user experience.

**Table 2.** Results of the Usefulness, Satisfaction, and Ease of Use questionnaire (n=10).

	Usefulness (n=56)	Ease of use (n=77)	Ease of learning (n=28)	Satisfaction (n=49)	Total (n=210)
Mean (SD)	44.20 (6.94)	63.90 (6.80)	23.90 (4.91)	42.90 (3.67)	174.90 (17.33)
Mean percentage (SD)	78.93 (12.39)	82.99 (8.84)	85.36 (17.53)	87.55 (7.50)	83.29 (8.25)
Median (IQR)	42.5 (13)	63 (9.8)	26 (8)	43.5 (5.8)	170 (28.8)

### Secondary Outcome: End User Feedback and Expert Observations

#### End User Feedback

First, at the start of the intervention period, many participants expressed concerns about their lack of knowledge and experience with video games and technology. They reported feeling unsure about their ability to complete or understand the intervention. However, by the end of the intervention period, this feeling of uncertainty disappeared as all participants reported that they felt like they were able to play and understand the game easily.

Second, difficulty perceiving the main character was often mentioned. Due to the color scheme used in the Ghostly game, participants experienced difficulties in discriminating the main character from the background. This often made it difficult to train efficiently and complete levels as they were unable to contract the muscle at the required time.

Last, participants who received Ghostly combined with BFR reported no discomfort during gameplay or postintervention, although participants mentioned feeling the cuff pressure during the intervention.

#### Expert Observations

In total, 5 experts observed the participants and collected their findings through field notes. First, experts noticed that older adults had difficulties with the reaction speed element integrated into the Ghostly game. As mentioned before, the participants are required to contract their muscles at the correct time for the main character to shoot or jump over obstacles. So, when users were unable to contract their muscles in time to avoid the obstacle, they were unable to complete the levels. In line with this, it was observed that when participants were unable to complete a level, the Ghostly game would restart the level. This led to a higher number of repetitions than initially prescribed and created a highly repetitive experience as participants continuously played the same level. Since participants were advised to complete 3 levels, after having to restart a level multiple times, it was sometimes unclear to participants how many levels still had to be completed before finishing the prescribed intervention session.

Second, with future unsupervised training in mind, the experts observed that participants understood the Ghostly game relatively quickly and understood the application of the sensors.

Next, experts noted that the difficulties experienced by the older adults in this study were specific to this population. They also observed variability within the group, with some participants struggling with fatigue, while others required additional time and support to learn and understand the Ghostly game. When expanding the Ghostly game beyond the population of older adults, the experts mentioned that a one-size-fits-all approach to game design will not fulfill the needs of all end users. Other patient populations might not experience the same difficulties and need different changes to enhance the usability of the game.

Last, the stability of game control occasionally posed challenges during the intervention. Specifically, 2 patients experienced difficulties with accurate registration of EMG signals by the Ghostly game during one of the intervention sessions, causing complications in completing certain levels. Nonetheless, despite these technical difficulties, all participants successfully completed the required intervention levels.

### Feasibility of the Intervention and Clinical Outcomes

#### Feasibility of the Intervention

After completion of data collection, all participants (15 out of 15 participants) completed all daily intervention sessions. Moreover, 13 out of 15 participants (86.67%) completed all measurements at baseline and before discharge from the hospital. Two participants were unable to complete the measurements at discharge due to feeling unwell at the time of measurement. This feeling was unrelated to the intervention. Last, no adverse events were recorded during or after the intervention. Based on these findings, it can be concluded that the study protocol is feasible.

#### Clinical Outcomes

**Figure 5.**
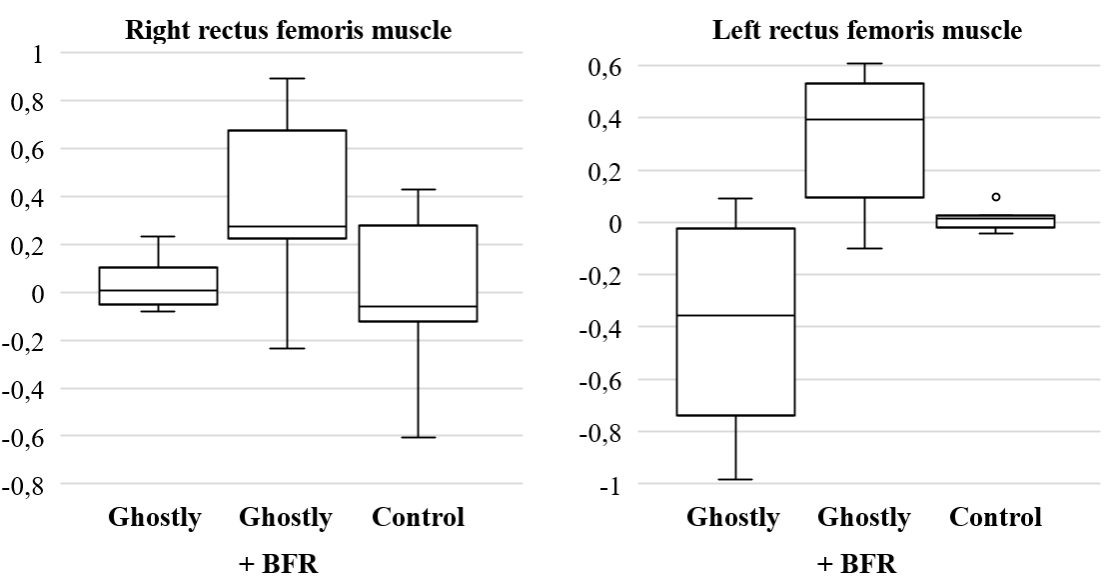
Boxplots of the mean difference in muscle cross-sectional area of the rectus femoris muscle for each group. The y-axis shows the mean difference in rectus femoris muscle cross-sectional area in cm. The x-axis shows the 3 intervention groups. BFR: blood flow restriction.

**Figure 6.**
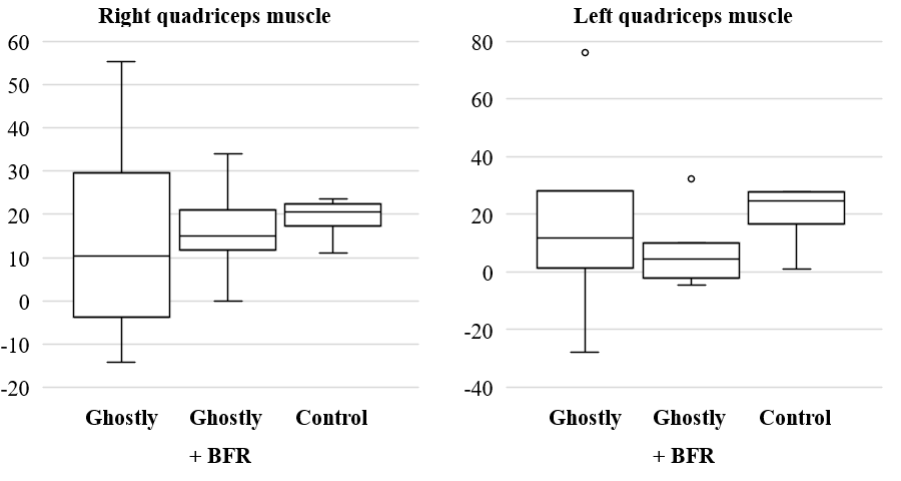
Boxplots of the mean difference in muscle strength of the quadriceps muscle for each group. The y-axis shows the mean change in quadriceps muscle strength in Newton. The x-axis shows the 3 intervention groups. BFR: blood flow restriction.

Clinical outcomes were assessed mainly focusing on the feasibility of the research protocol for a future fully powered RCT. Boxplots were used to visualize the distribution of the data on rectus femoris CSA (Figure 5) and quadriceps muscle strength (Figure 6) of both legs. After the intervention, the Ghostly + BFR group showed the largest mean increase in CSA of 0.34 cm (95% CI 0.08 to 0.59) for the rectus femoris muscle compared with other 2 groups. For muscle strength, a mean difference of 12.18 N (95% CI 2.43 to 21.92) was found for the quadriceps muscle in the Ghostly + BFR group. This was similar to the mean difference in muscle strength of both other groups, with 16.71 N (95% CI –12.09 to 45.51) for the quadriceps muscle in the Ghostly group, and 19.29 N (95% CI 11.70 to 26.88) for the quadriceps muscle in the control group. Results on rectus femoris muscle thickness revealed the largest increase in the Ghostly + BFR group with a mean difference of 0.01 cm (95% CI –0.12 to 0.13). Lower limb subcutaneous fat showed the largest mean difference in the control group, with a decrease of 0.02 cm (95% CI –0.07 to 0.10). Last, the largest mean difference in lower limb lean mass was found in the Ghostly + BFR group (mean difference: 1.83 kg, 95% CI –0.12 to 3.77). A full overview of the mean differences of all outcomes can be found in Table 3.

**Table 3.** Clinical outcome measures of participants separated into the 3 intervention groups. Mean difference and 95% CI of muscle characteristics for each group

	Ghostly (n=4)		Ghostly + BFR^a^ (n=5)		Control (n=4)	
	Δ^b^	95% CI	Δ	95% CI	Δ	95% CI
30-second sit-to-stand (repetitions)	2.00	–0.25 to 4.25	1.00	–0.96 to 2.96	1.00	–0.30 to 2.30
Quadriceps muscle strength (Newton)	16.71	–12.09 to 45.51	12.18	2.43 to 21.92	19.29	11.70 to 26.88
Rectus femoris muscle thickness (cm)	–0.05	–0.18 to 0.08	0.01	–0.12 to 0.13	–0.03	–0.09 to 0.04
Rectus femoris muscle cross-sectional area (cm²)	–0.18	–0.53 to 0.17	0.34	0.08 to 0.59	–0.00	–0.20 to 0.19
Lower limb subcutaneous fat (cm)	0.01	–0.07 to 0.09	–0.03	–0.09 to 0.03	0.02	–0.07 to 0.10
Lower limb lean mass (kg)	0.84	–1.46 to 3.13	1.83	–0.12 to 3.77	0.94	–1.65 to 3.53

a BFR: blood flow restriction.

b Δ: mean difference from T0 to T1.

## Discussion

### Principal Findings

This mixed method feasibility study investigated the usability and feasibility of an EMG-driven exergame combined with BFR in relation to strength training in hospitalized older adults. Currently, no other studies have combined an EMG-driven exergame with BFR, highlighting the innovative aspect and relevance of our results in the field of exergames. This study is an early part of a larger iterative process of user-centered technology development. The main focus of this study was to gather information on the user experience of older adults playing the Ghostly game as a stand-alone added therapy or in combination with BFR, and how the game can be updated to further adhere to the needs and recommendations of the end users while simultaneously adhering to evidence-based guidelines for strength training. Second, clinical outcome measures were assessed to test the feasibility of the study protocol in preparation for a future larger RCT aimed at investigating the clinical effectiveness of the Ghostly game.

The results of the user experience questionnaire indicate overall high satisfaction with the Ghostly game, as well as good scores on usability and ease of learning. When only focusing on the user experience results from the Ghostly + BFR group, high scores remained, implying that BFR does not adversely influence user experience. These findings strengthen the evidence for the Ghostly game combined with BFR as a viable rehabilitation approach in this target population. In this literature, only one study by Mousavi et al [43] investigated the effects of BFR in combination with a boxing exergame for the Xbox 360 Kinect, on enjoyment and energy expenditure. However, this study reported decreased, although not significant, perceived enjoyment and no difference in energy expenditure in 14 healthy participants after a single session of the boxing exergame combined with BFR compared with a single session with the exergame alone [43]. This hiatus in literature further highlights the relevance of this study and the need for more research into BFR combined with exergames. A potential explanation for the limited implementation of exergames and BFR could be the concern of increased hemodynamic stress and disturbances in local blood flow [44], however, correct implementation of BFR has been shown to present no greater risk than conventional exercise [45].

### Comparison to Prior Work

Further expanding on the user experience while training with exergames, our results are in line with literature showing that exergames have a beneficial effect on intrinsic motivation and thus might increase therapy adherence [46-48]. As aforementioned, Garcia-Hernandez et al [23] reported significantly higher intrinsic motivation in patients who trained with an EMG biofeedback exergame compared with patients performing conventional therapy. The exergames group showed higher mean scores for interest and enjoyment, as well as perceived competence [23]. Similarly, Fitzgerald et al [49] reported high scores on interest and enjoyment in healthy adults who performed 12 sessions of postural stability training with a wobble board-based exergame. These findings highlight the potential of exergames to motivate patients to remain active during and after rehabilitation. As revealed by Ong et al [50], exergames can prove to be an efficient tool for rehabilitation, but there is a need for end user perspectives in the development of evidence-based exergames to ensure sufficient implementation. A user-centered design approach is required to support the development of rehabilitation technologies that maximize both user acceptance and functional outcomes [51].

Data from end user feedback and expert observations highlighted a few key elements that should be adjusted to further enhance the user experience of the end users such as a change of color contrast, different level difficulties, etc. Expanding on these findings, the noticeable decline in color contrast perception experienced by older adults due to perceived yellowing of the human lens warrants the need for a change in color contrast in the Ghostly game [52]. Different color combinations produce varying degrees of difficulty in perceiving contrast. For example, the contrast between green and blue is often more difficult to perceive in older adults [53,54]. Moreover, this decline in contrast perception strongly influences reaction time as older adults need more time to detect, discriminate, and recognize objects in the visual field [54,55]. Noticeably, the main color scheme of the Ghostly game is blue and green, which potentially explains some of the difficulties participants experienced while playing the Ghostly game. Research recommends color combinations with medium to long wavelengths, such as yellow, orange, and red, as these are easier to perceive, or to implement a large contrast in lightness when using blue or green to ensure sufficient contrast perception [54].

Furthermore, as people grow older, there is a generalized slowing in information processing, with a more specific increase in the amount of time necessary to make a correct judgment about visual stimuli, also known as visual processing speed [56]. Older adults often experience difficulties in tasks that involve visual clutter, secondary task demands, and time-sensitive responses [56], all of which are incorporated into the Ghostly game. With this in mind, it is important to add changes to the Ghostly game that take into account this decline in information processing speed by creating levels that limit the need for quick processing of visual information. Nonetheless, the cognitive-motor dual-task aspect of the Ghostly game should not be fully removed, as dual-task training has positive effects on cognitive and physical function [57]. A review by Joubert and Chainay [58] reported a positive effect of cognitive-motor dual-task training on cognitive functioning. Similarly, a study by Uematsu et al [59] on the effect of a cognitive-motor dual-task standing balance exergame reported a beneficial effect of exergame balance training on one-leg standing time, functional reach distance, and hip and knee extensor strength in 25 healthy older adults.

Focusing on the secondary outcomes of this feasibility study, descriptive statistics revealed very limited effects on clinical outcomes. Nonetheless, these outcomes are relevant in hospitalized older adults. Within this population, muscle weakness and muscle mass loss due to inactivity can lead to a functional decline and loss of independence, which is not directly related to the condition for which they were admitted, also defined as hospital-associated disability [7]. Due to the short intervention period, no changes in clinical parameters were expected. Literature has shown that early changes in muscle strength and mass can occur after 2 to 4 weeks of training, but the adaptations after resistance training are generally evident after 8 to 12 weeks of training [60]. The early adaptations can be explained by neuromuscular and connective tissue adaptations, and edema-induced swelling of the muscle [60,61]. Nonetheless, as described in the results, all interventions and outcomes were feasible in relation to a future larger RCT testing the effectiveness of the Ghostly game.

### Future Directions

With future development and research in mind, an updated version of the Ghostly game combined with BFR might prove to be a relevant tool for rehabilitation to promote the Aging in Place framework proposed within the Healthy Aging model of the World Health Organization [62,63]. Aging in Place is defined as the ability of older adults to remain in their community during later life to preserve a sense of connection, security, and familiarity, as well as their sense of identity and autonomy [62,63]. As stated earlier, exergames have proven to enhance motivation and willingness to exercise in older adults, even when unsupervised [46-48]. Thus, the Ghostly game can provide opportunities for older adults living independently or in community homes and residential settings who are unable to train in a high-load, more dynamic way to remain active in a motivating and challenging manner. Moreover, implementing BFR into the Ghostly game in a comprehensive manner ensures evidence-based training at an intensity that is beneficial for rehabilitation.

### Strengths and Limitations

To date, no other studies combining an EMG-driven exergame with the strength training principles of BFR training in hospitalized patients have been found in the literature, highlighting the relevance of our results in the field of exergame research. This combination of evidence-based game design and the already-established effectiveness of BFR training in older adults maximizes the potential for rehabilitation in hospitalized older adults. Second, another strength of this study is the mixed methods nature of the study design, which provides a comprehensive understanding of both the user experience of the participants while using the Ghostly game, as well as the clinical effects of the Ghostly game for rehabilitation. Third, the implementation of a wide variety of target groups (ie, patients and health care professionals) in the investigation of the user experience ensures an all-encompassing vision of the needs and recommendations, without being limited to the expertise of one specific demographic.

Nonetheless, this study has a few limitations that warrant caution when interpreting the results. First, it is important to note that the intervention period of this feasibility study was short, ranging from 3 to 5 sessions. However, as the main goal of this study is to gather information on user experience, 3 to 5 sessions were sufficient to gather reliable user experience data from all the participants. Second, the study sample involved 15 participants, with 10 participants training with the Ghostly game. This small sample may restrict the trial’s ability to detect meaningful effect sizes, but it is sufficient to gather user experience data. Last, as mentioned in the results, game control was not always stable while completing the intervention due to inaccurate registration of EMG signals. It seems plausible to hypothesize that the EMG signal issues may partly result from variations in participants’ subcutaneous fat thickness. Subcutaneous fat is known to reduce the EMG amplitude above active muscles [64]. Kuiken et al [64] reported a 30%‐80% reduction in EMG amplitude with fat layers of 0.3‐0.9 cm. In our study, the average baseline subcutaneous fat was 0.85 cm (SD 0.52 cm), with 4 participants exceeding 0.9 cm, of which only one experienced issues. However, by implementing an assessment level before the Ghostly game is played, inaccurate EMG signals are filtered, and potential signal issues could be identified before the start of the intervention. When signal issues occurred, the supervising researcher addressed them by disconnecting and reconnecting the EMG sensors and restarting the assessment procedure until stable EMG signals were observed. Additionally, observations regarding EMG signal stability were documented to inform future development of the Ghostly game.

### Conclusions

As part of a larger iterative development process, this mixed method randomized feasibility study investigated the user experience and feasibility of an evidence-based exergame, the Ghostly game, as a stand-alone added therapy or in combination with BFR for rehabilitation of bedridden hospitalized older adults. The results revealed a high overall level of satisfaction and usability of the Ghostly game. Furthermore, analysis of the USE questionnaire demonstrated good scores when only focusing on the Ghostly + BFR group. Our findings also highlighted important changes required to maximize user experience based on expert opinions and participants’ recommendations. Since this study is part of a larger user-centered design approach, the results further emphasize the necessity of incorporating all end users in the development of rehabilitation technology with the aim of maximizing the functional capabilities of the users, as well as maximizing user experience. Additionally, this study shows the potential of exergames combined with BFR to maximize clinical outcome effects based on state-of-the-art evidence on strength training. In regard to feasibility, it was found that all outcomes and procedures were feasible for a larger RCT testing the effectiveness of the Ghostly game in combination with BFR.

## Supplementary material

10.2196/69400 Multimedia Appendix 1 Template for intervention description and replication checklist for the 3 intervention arms.

10.2196/69400 Multimedia Appendix 2 Results of the Usefulness, Satisfaction, and Ease of Use questionnaire for each intervention group.

10.2196/69400 Checklist 1 The CONSORT-eHealth checklist (V 1.6.1).

## Abbreviations

ALM: appendicular lean mass
AOP: arterial occlusion pressure
BFR: blood flow restriction
CSA: cross-sectional area
EMG: electromyography
MVC: maximal voluntary contraction
RCT: randomized controlled trial
USE: Usefulness, Satisfaction, and Ease of Use
