# Supplementary material for: Insights on the User Experience and Feasibility of an Electromyography-Driven Exergame Combined With Blood Flow Restriction for Strength Training in Hospitalized Older Adults: Mixed Methods Randomized Controlled Feasibility Study
Source: JMIR Serious Games. 2025 Jun 10;13:e69400. doi: 10.2196/69400 (PMC12173152; doi:10.2196/69400)
Supplement: Multimedia Appendix 1 [file games-v13-e69400-s001.docx]

| **Appendix 1:** Template for intervention description and replication checklist for the three intervention arms. | | | |
| --- | --- | --- | --- |
| Brief name | Ghostly | Ghostly + BFR | Control |
| Why | Exergames combine exercise with the motivational aspect of interactive video games. The Ghostly game addresses both, the need for low load strength training and the potential lack of motivation for exercise. | Low load BFR training is proven relevant in older adults. The combination of the motivational aspects of the Ghostly game with the training effects of BFR might prove relevant in increasing strength in older adults while exercising with the Ghostly game. | Low load isometric exercises can be beneficial in bedridden patients who are unable to exercise upright in a functional high load manner. |
| What | The Ghostly game is an EMG-based exergame in which the main character is controlled through muscle contractions measured by two surface EMG-sensors placed on the skin over the targeted muscles. All levels were designed to require at least twelve contractions with each limb . Since the participants are asked to complete three levels, they perform three sets of twelve repetitions.  The priority is training at one’s personal maximal capacity, so the Ghostly game incorporates a baseline assessment level in which the MVC of the user is measured. The surface EMG sensors are applied over the belly of the target muscles by the researcher. First, the assessment level needs to be completed to set the intensity at which the participants have to contract the muscles to play the game. After completion of the assessment level, a training level is selected and the Ghostly game is played. | The second therapy arm of this study involves the addition of BFR therapy to the Ghostly game. BFR entails the partial occlusion of blood flow towards the exercising limb, which results in a partial inflow of arterial blood and complete restriction of venous outflow. The BFR system used in this study is the SmartCuffs pro system (Smart Tools, USA). The cuffs used in this study had a width of 10 cm and length of 58 cm. | Isometric strength training exercises dose-matched to the Ghostly game are provided to the participants as an added therapy modality on top of the conventional physiotherapy provided in the geriatric ward of the hospital. |
| Who provided | All interventions are provided under supervision of the researchers. | | |
| How | The Ghostly game is provided to the participants as an added therapy modality on top of the conventional physiotherapy provided in the geriatric ward of the hospital. On a daily basis, the researchers visit the participants in their room and set up the game while the participant remains in bed. The surface EMG sensors connected to the game are placed over the middle of the muscle belly of the m. rectus femoris using double sided adhesive tape. After the surface EMG sensors are applied and the Ghostly game is started, the assessment level within the game is completed. This assessment level consists of three maximal contractions, with each contraction lasting three seconds and with 90 seconds of rest between each contraction. After the first maximal contraction, a MVC value is set matching the intensity of this first contraction. If subsequent contractions exceed the MVC value, the value is raised accordingly. Based on this MVC value, the intensity at which the user must contract to control the main character is set to match an intensity of 75% of MVC. Following the assessment level, a training level is selected and the participants start playing under supervision of the researchers. The intervention ends after the participant completes three training levels. | As an added training stimulus to the Ghostly game, BFR is applied to the participant who remains in bed. Before the application of the surface EMG sensors on the participants, the BFR smart cuffs are placed over the proximal part of both lower limbs to partially occlude the blood flow towards the quadriceps muscles. To achieve the desired level of occlusion to the trained muscle, a percentage of AOP is used measured by the SmartCuffs pro system. AOP is defined as the amount of pressure required to completely cease arterial blood flow towards the limb. To determine AOP, the cuffs are filled with air until a pressure is reached in which no arterial blood pressure is registered by the SmartCuffs pro system. This pressure is then set as the AOP and all air within the cuffs is automatically released. Afterwards, the cuffs are re-inflated until 50% of the personalized AOP is reached. Once the cuffs are inflated, the surface EMG sensors are installed on the participant and the game is played. After completion of three levels in the Ghostly game the researchers release the pressure from the cuffs and take off the cuffs. | On a daily basis, the researchers visit the participants in their room and perform three sets of twelve isometric contractions of the quadriceps muscle while the participant remains in bed. |
| Where | All interventions were performed at the geriatric ward of the University hospital of Brussels. | | |
| When and How much | Participants received daily supervised interventions on top of their conventional physiotherapy starting after baseline measurements until discharge from the hospital. | | |
| Tailoring | The assessment level incorporated in the game ensures that participants train at a personalized intensity. This assessment level needs to be completed at the start of every intervention session. | On top of the personalized training intensity of the Ghostly game, a personalized AOP is used to determine the occlusion pressure of the BFR cuffs. | The control intervention was not personalized. |
| How well | With the goal of testing feasibility of the research protocol, intervention adherence rate was documented. Participants were expected to complete daily intervention sessions starting after baseline measurement until discharge from the hospital. All participants completed all intervention sessions (100% intervention adherence). | | |
| Abbreviations: AOP= arterial occlusion pressure, BFR= blood flow restriction, EMG= electromyography, MVC= maximal voluntary contraction | | | |
