# Supplementary material for: Insights on the User Experience and Feasibility of an Electromyography-Driven Exergame Combined With Blood Flow Restriction for Strength Training in Hospitalized Older Adults: Mixed Methods Randomized Controlled Feasibility Study
Source: JMIR Serious Games. 2025 Jun 10;13:e69400. doi: 10.2196/69400 (PMC12173152; doi:10.2196/69400)
Supplement: Multimedia Appendix 2 [file games-v13-e69400-s002.docx]

| **Appendix 2:** Results of the Usefulness, Satisfaction and Ease of use questionnaire for each intervention group | | | | | | |
| --- | --- | --- | --- | --- | --- | --- |
|  | **Ghostly (n= 5)** | | | **Ghostly + BFR (n= 5)** | | |
|  | Mean (±SD) | Mean (%) | Median | Mean (±SD) | Mean (%) | Median |
| Usefulness (/56) | 46.20 ± 6.37 | 82.50% | 44 | 42.20 ± 6.91 | 75.36% | 40 |
| Ease of use (/77) | 67 ± 5.80 | 87.01% | 64 | 60.80 ± 6.31 | 78.96% | 60 |
| Ease of learning (/28) | 25.20 ± 2.99 | 90% | 26 | 22.60 ± 5.99 | 80.71% | 26 |
| Satisfaction (/49) | 43 ± 4.20 | 87.76% | 44 | 42.80 ± 3.06 | 87.35% | 43 |
| Total (/210) | 181.40 ± 16.76 | 86.38% | 171 | 168.40 ± 15.34 | 80.19% | 160 |
| Abbreviations: SD= standard deviation | | | | | | |
